# Theory and Application of Magnetic Flux Leakage Pipeline Detection

**DOI:** 10.3390/s151229845

**Published:** 2015-12-10

**Authors:** Yan Shi, Chao Zhang, Rui Li, Maolin Cai, Guanwei Jia

**Affiliations:** 1 School of Automation Science and Electrical Engineering, Beihang University, Beijing 100191, China; zhangchaobuaa3xi@163.com (C.Z.); kjlirui@petrochina.com.cn (R.L.); caimaolin@gmail.com (M.C.); jiaguanwei@126.com (G.J.); 2 PetroChina Pipeline Company, Langfang 065000, China

**Keywords:** in-line inspection, magnetic flux leakage detection, pipeline, review

## Abstract

Magnetic flux leakage (MFL) detection is one of the most popular methods of pipeline inspection. It is a nondestructive testing technique which uses magnetic sensitive sensors to detect the magnetic leakage field of defects on both the internal and external surfaces of pipelines. This paper introduces the main principles, measurement and processing of MFL data. As the key point of a quantitative analysis of MFL detection, the identification of the leakage magnetic signal is also discussed. In addition, the advantages and disadvantages of different identification methods are analyzed. Then the paper briefly introduces the expert systems used. At the end of this paper, future developments in pipeline MFL detection are predicted.

## 1. Introduction

### 1.1. The Significance of Pipeline Defect Detection

Nowadays, petroleum and natural gas are key energy and chemical raw materials, which have a pivotal function in the life of people, industrial and agricultural production and national defense [1]. It is well known that most safest and effective way to transport oil-gas is to employ a pipeline system. However, most pipelines are buried underground, where they are easily affected by humidity or pressure and are prone to deformation and corrosion. Any metal losses or small defects in pipelines could cause serious accidents [2,3]. 

The pipeline industry in China has developed along with the oil industry. After more than 40 years, the gap between China and the world’s developed countries has gradually shrunk, especially in the areas of pipeline engineering design technology, construction level, operation management and maintenance [4,5], but China’s pipeline industry started relatively late and has developed slowly, and there are many considerable gaps in the pipeline coverage, service range, technical equipment and so on. Oil and gas companies have a vast amounts of facilities and equipment. To ensure safe operation is paramount, and there are considerable maintenance methods that can be adopted. The cost for repairs or replacement of subsea pipelines is much higher than for onshore pipelines [6]. The Baltic Sea pipeline will be an important contribution to the long-term security of supply and the energy partnership between the European Union and Russia, but lots of work is needed to protect the pipelines. The safety of deepwater risers is essential for sustainable operation of offshore platforms, which need to increase the accuracy of damage detection and fatigue estimation. Some spiral weld defects exist on certain old and long pipelines of PetroChina (Beijing, China). These defects are formed due to a lack of penetration and fusion during pipe manufacturing. Every year the cost of pipeline maintenance of China is as much as several hundreds of millions Yuan, and there is an increasing trend. Restricted by detection technology and means, pipeline detection is blind, which leads to a waste of manpower, materials and financial resources. All this puts more pressing requirements on pipeline detection [7,8,9,10].

### 1.2. The Brief Introduction of MFL

**Figure 1:**
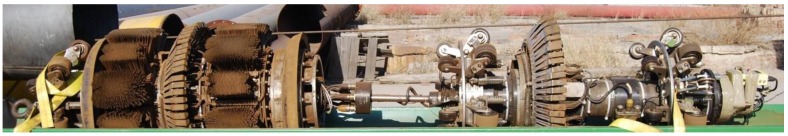
A MFL tool, comprising three main sections: a drive section at the front of the tool, a central magnetizer section and a data logger situated towards the rear of the tool.

Nondestructive testing techniques have been widely used for evaluating the condition of pipelines. Common methods include ultrasonic inspection techniques, eddy current inspection techniques, ray inspection techniques, penetration inspection techniques and magnetic flux leakage inspection techniques. Each method has its advantages and limitations. Because of the special feature of the long nature of oil pipeline inspections, these are carried out from the interior of the pipe. The ultrasonic inspection, eddy current inspection and magnetic flux leakage inspection techniques are common methods. Magnetic flux leakage and the ultrasonic inspection technique are the most frequently used for evaluating the integrity of pipelines. However, due to various factors and impact of the constraints, both of their results are uncertain, and the results need to be quantified and evaluated. Magnetic flux leakage is the most widely used method which can detect cracks in both the axial and circumferential directions, although it is susceptible to the pipe wall and other factors. As noted, magnetic flux leakage (MFL) techniques have evolved in the pipeline inspection industry since the 1960s [11,12,13,14,15]. In the initial stage, the sensors widely used magnetic powder, which displayed the results by piling up. This method is intuitive, simple, has high sensitivity, and has been widely used in industry. With the development of the semiconductor electronics industry, magnetic sensors have made great progress, which has removed the limitation of measuring tools to magnetic powder. Figure 1 shows a MFL tool.

Magnetic flux leakage inspection does not need pre-processing and the signals are easy to detect. Online detection can be easily carried out and a high degree of automation can be implemented. Besides, it can detect many types of defects. For example, surface defects, stomata, scars, shrinkage cavities, corrosion pitting and so on. It can not only examine the internal surface for defects, but also external surfaces [16]. The requirements for the detection environment is not high, and they are unaffected by the transportation medium. All of these advantages make magnetic flux leakage inspection the most popular method. During the inspection process, a MFL_PIG is sent through the buried pipe to perform the pipeline inspections. If you're standing near a pipeline where a MFL_PIG is working, vibrations can be felt as pigs move through the pipeline, that’s why magnetic flux leakage detectors were called intelligent pigs [17,18,19].

Magnetic flux leakage inspection began to be widely used from the beginning of the 50s in the twentieth century. From then on, it has evolved from the qualitative identification of defects to a quantitative analysis phase [20]. Although some encouraging theoretical and experimental achievements had been obtained, it is still imperfect and incomplete. The main limitations can be listed as follows [21,22]:

1. So much qualitative analysis of the signal is needed that MFL is hardly applied in practice, because the actual complicated working conditions can’t match the laboratory conditions.
2. It is quite sensitive to the running speed of the vehicle.
3. The pipe wall must achieve complete magnetic saturation.
4. It has a strong capability to detect large area while it is limited to the material surface and near surface; the detection of axial narrow and long defects is restricted.
5. The probe is susceptible to the pipe wall, and its anti-interference ability is poor. When the materials used in the pipeline are mixed with impurities, there will be false data.
6. The quantitative theory of defects needs to be further studied. There is no one-to-one correspondence between the shape of the defect and the signal characteristics of the detection.
7. The height of the defect sometimes depends on the experience of the operator.

## 2. Principle, Model and Influence Parameter

### 2.1. Principle of Magnetic Flux Leakage Detection

**Figure 2:**
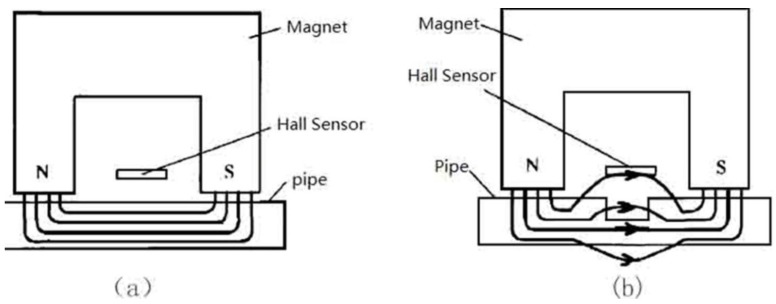
Principle of magnetic flux leakage detection. (**a**) Pipe without metal loss; (**b**) Pipe with defect.

The basic idea of magnetic flux leakage inspection is that the ferromagnetic material is magnetized close to saturation under the applied magnetic field. The basic principles are shown in Figure 2. If there is no defect in the material, most magnetic flux lines will pass through the inside of the ferromagnetic material; if not, because the magnetic permeability of the defect site is much smaller than that of the ferromagnetic material itself, magnetic resistance will increase in the defect area, so the magnetic field in the region is distorted. Magnetic flux lines will be bent, some will leak out of the material surface, and a magnetic leakage field will form at the defect area [23,24,25]. By using magnetic sensitive sensors to detect the magnetic leakage field, the corresponding electrical signals can be obtained. Then the detected signals are analyzed so that the status of the defect can be determined. Defects caused by corrosion can appear in the interior and exterior surfaces of the pipeline, and magnetic flux leakage pipeline detection can detect them, but it cannot distinguish between internal and external defects. Therefore, a classification approach based on support vector machines (SVM) is presented to achieve defect discrimination [26].

**Figure 3:**
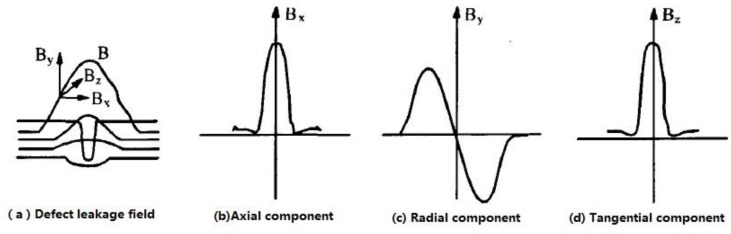
Defect leakage field and each component.

When there is a defect in the pipeline, the defect leakage field is generated (Figure 3a), and the vector distributions of each component are shown in Figure 3b–d. The horizontal axis represents the width of the defect; the vertical axis represents the intensity of the magnetic induction.

Magnetization of the pipe wall is needed before the experiment. The methods for this can be divided into AC magnetization, DC magnetization and permanent magnet magnetization [27,28].

1. AC magnetization. It can be used to detect a workpiece on which surface is rough, but AC magnetic field easily produces skin effects and eddy currents, and the depth of magnetization decreases with the increase of current frequency. Therefore, this method can only detect surface and near surface defects, but the intensity of AC magnetization is easy to control, the magnetic structure is simple, and the cost is low.
2. DC magnetization. DC magnetization is divided into DC pulsating current and DC constant current magnetization, the latter being simpler than the former in structure; however, the excitation current is larger. It can detect more than 10 mm deep surface defects, and magnetization can be easily adjusted by controlling the size of the current, but it is difficult to achieve larger magnetizations, and demagnetization is needed every time it is used.
3. Permanent magnet magnetization. This uses a permanent magnet as the excitation source. It has the same characteristics as DC magnetization, but the adjustment of intensity is less convenient than in DC magnetization. Permanent magnets can be made with permanent ferrite, aluminum and nickel cobalt permanent magnet materials and rare earth permanent magnet materials. Especially rare earth permanent magnet materials, because of the high energy nature, small volume and no need or electricity, have been well applied in magnetic flux leakage detection.

**Figure 4:**
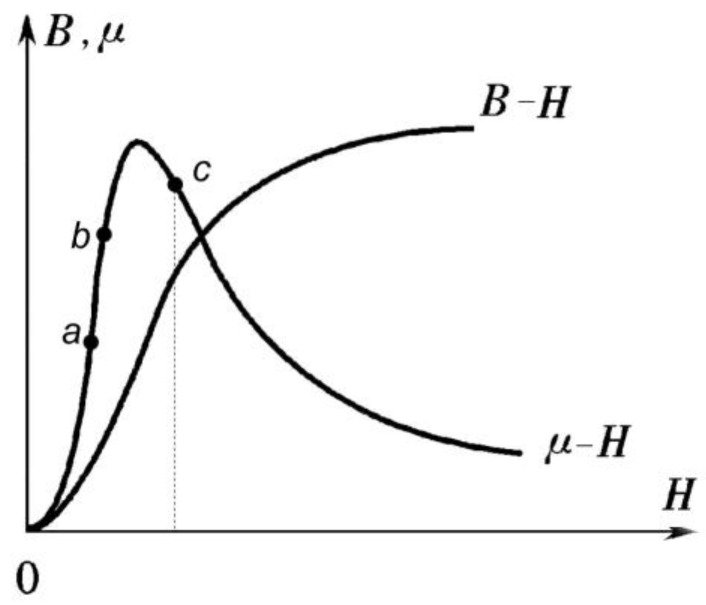
Relationship between magnetic conductivity μ, magnetic field strength H and magnetic flux density B.

In magnetic flux leakage detection, although the detection purposes are different, the intensity of magnetization should first be chosen in the case that leakage magnetic field can be detected. The magnetic conductivity changes with the magnetic field strength as shown in Figure 4. In the “a–b” section, the magnetic flux intensity is increased due to the presence of defects, thus the magnetic conductivity is increased. This is bad for detection. Therefore, the magnetic field strength is selected while μ declines most rapidly. For example, one would select the “c” point in the Figure 4. Other factors need to be considered in actual situations, such as signal to noise ratios and the economic performance of the detection device.

### 2.2. Analytical Model of the Magnetic Leakage Field

**Figure 5:**
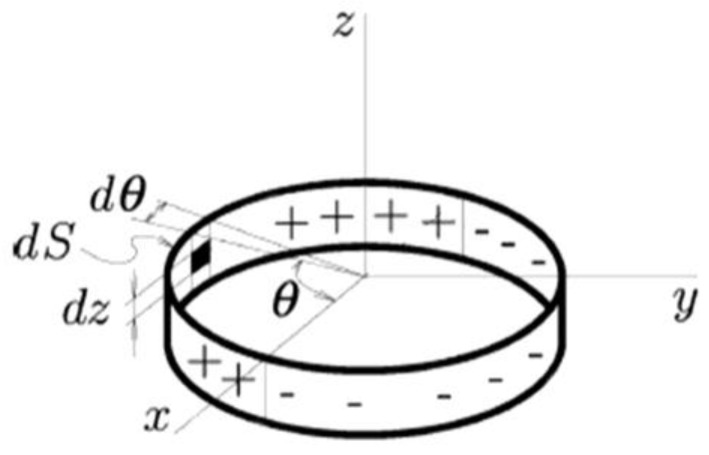
Dipolar representation of a cylindrical hole defect.

The magnetic dipole model was the first theoretical model which related the shape of a defect with the magnetic field strength, the magnetic permeability of the material and the leakage magnetic field. For defects such as holes and pits, an equivalent point dipole model can be used to simulate them, as shown in Figure 5; defects such as scratches could be simulated using an equivalent linear dipole model; defects such as cracks can be in a first approximation compared to an infinitely long rectangular slot, then simulated using an equivalent surface dipole model.

Although the application of this model greatly simplifies the analysis difficulties it establishes a two-dimensional model which can’t completely reflect the real situation; besides, it is assumed that the permeability of the material is linear, so there are some errors in the theoretical and experimental results [28,29,30]. The results of the model can only explain simple and regular defects.

The development of modern electromagnetic theory deepened people’s understanding, and the related electromagnetic field theory is based on the Maxwell equation which is a vector partial differential equation. In three-dimensional space, using the boundary conditions for solving equations by analytic methods is almost impossible [31,32,33,34,35]. Therefore, a numerical calculation method is usually used. The finite element method is the most widely used numerical calculation method. By establishing an equivalent energy functional for the vector partial differential equation of electromagnetic field, and in the approximate function space, the minimum value of the function is obtained. In this way, it transforms the complex electromagnetic field vector partial differential equation into algebraic equations solving the unknown vector magnetic in discrete areas of each network [36,37,38,39,40].

In 1975, Lord and Hwang first introduced the finite element method into the calculation of the magnetic leakage magnetic field, which made the theoretical research progress greatly. Through the study of the influences of different shapes, angles, depths and widths of defects’ on the leakage magnetic field, they pointed out that to solve the problem of the complex shape of magnetic flux leakages, numerical calculation is the only feasible method. Atherton and Bruder did a lot of work in the numerical calculation of leakage magnetic fields. The inside and outside tube wall defects were calculated, a two-dimensional model of leakage magnetic field distribution was established, and the calculated results and experimental results were compared and found to be quite consistent; they also applied the finite element method to the analysis and optimization of the magnetization of the wall and used the calculated results as the basis choice of the magnetization method [41]. Yan and others also applied the finite element method for defect leakage magnetic field models. Using a feedback iteration method they analyzed the errors of the model and then did a simulation for natural gas transportation pipeline appearance defects. They also put took some influencing factors of leakage magnetic field and magnetic flux leakage signals into consideration, such as the magnetic pole movement speed in the pipe, pipeline magnetization, pipe material, *etc*.

### 2.3. Defect Shape Influencing Parameters

The leakage magnetic field will be affected by many factors, including tube wall magnetization and residual magnetism, the pipeline material (electrical and magnetic conductivity), magnetic field coupling circuits (such as steel brushes used for magnetic coupling to the pipe wall) and the distance between the magnetic poles; speed of movement of the detector in the pipeline; pipeline pressure variations, *etc.* For example, lift-off caused by welds and debris is an insidious problem that affects the acquired magnetic flux leakage data in pipeline MFL inspection [42,43,44]; an increase in the speed of the detector can cause the leakage flux of the defect to be obviously reduced, and the speed changes should be considered when higher defect detection accuracy is needed; the greater the thickness of the tube wall, the stronger the external magnetic field required to achieve saturation magnetization. In an external magnetic field of invariant intensity, tube wall thickness changes and pipe wall magnetic field intensity and magnetic induction intensity are contrast to the linear relationship, that is to say, when the tube wall is thicker, the tube wall of the magnetic induction intensity is smaller; when the wall is magnetized, the magnetic leakage field can be generated at the defect, which depends on the magnetization of the tube wall [45,46,47]. If the magnetization intensity is not enough, then defects of thin wall thickness may carry all of the flux, so you do not have leakage flux. At the same time, magnetization cannot be oversaturated, because it will not only increase the air coupled magnetic field, resulting in a defect signal to noise ratio decrease, but also heat the pipe wall and reduce defect resolution ability. Remanence is used for magnetic flux leakage testing and on the tube wall in the residual magnetic field, in the pipe wall magnetic level is low or medium, it will reduce the pipe wall of the magnetic induction intensity, thus affecting the detection and quantification of defects.

**Figure 6:**
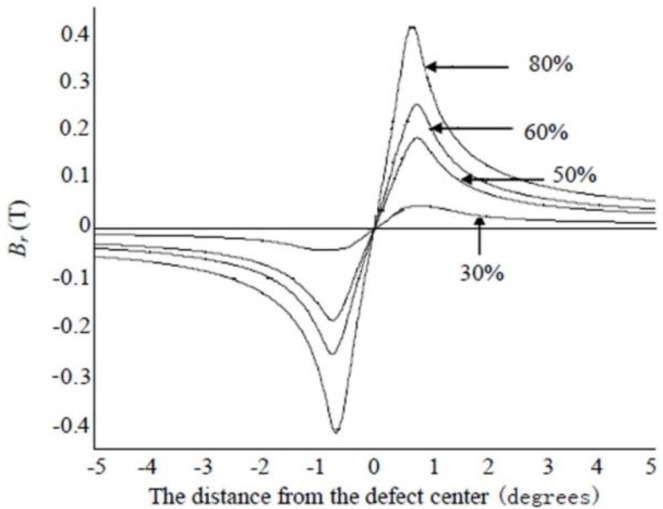
Radial MFL signals with different depth.

**Figure 7:**
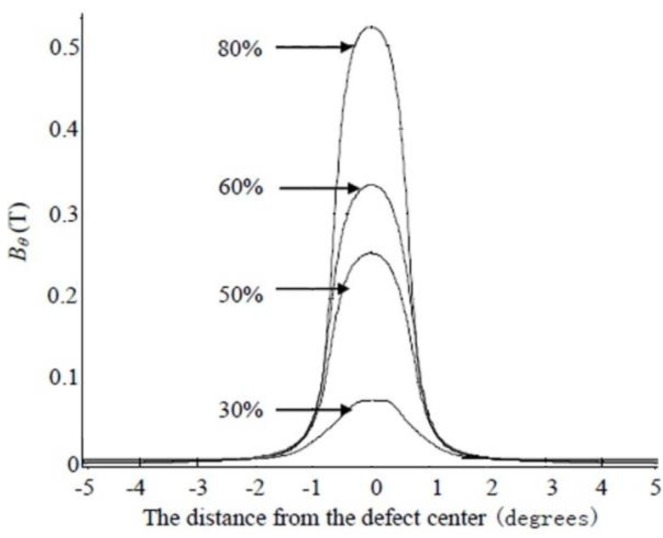
Circumferential MFL signals with different depth.

**Figure 8:**
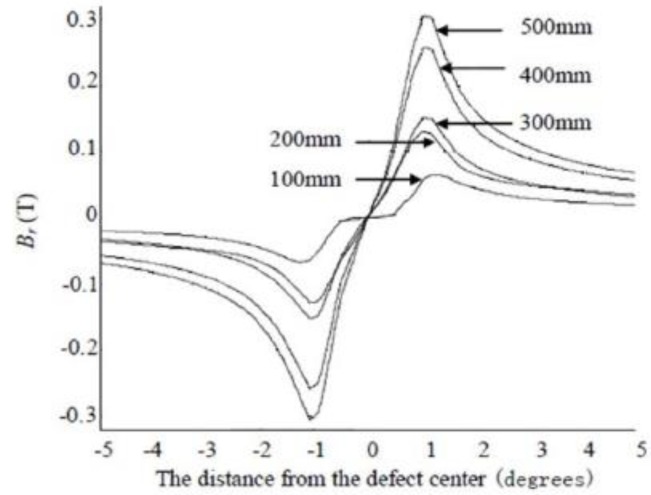
Radial MFL signals with different length.

**Figure 9:**
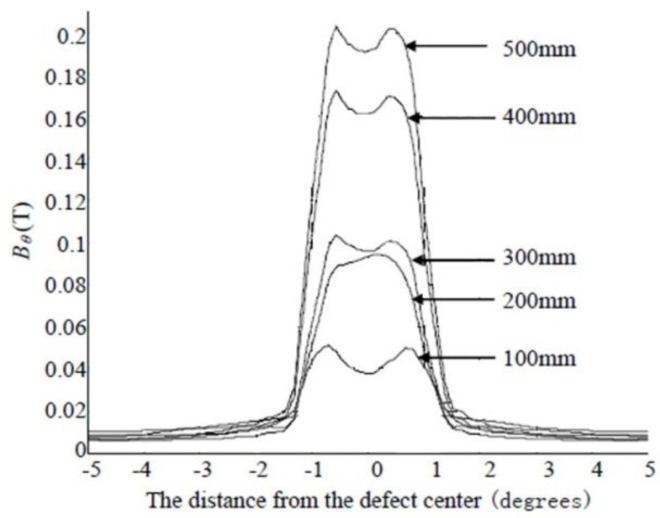
Circumferential MFL signals with different length.

Figure 6 and Figure 7 respectively show the radial and circumferential components of MFL signals with different depth. Figure 8 and Figure 9 respectively show the radial and circumferential components of MFL signals with different length.

In short, in order to improve the detection accuracy, high strength of magnetization of the magnetic field strength should be chosen to ensure that the wall achieves moderate saturation; the appropriate wall thickness and magnetic pole spacing should be selected; the detector should be moving to ensure the wall reaches saturation magnetization and so on [48,49,50,51].

## 3. Measurement and Processing

### 3.1. Measuring Methods and Sensors

With the development of electronic technology, computer technology and sensors, magnetic field measurement methods have been developed greatly. The main methods include [52]:

1. Electromagnetic induction method. Based on Faraday's law of electromagnetic induction, it is one of the most basic magnetic measurement methods. It can measure DC, AC and pulsed magnetic fields. Measuring instruments usually use induction coils, including impact galvanometers, flux meters electronic integrators and vibrating coil magnetometers [53,54].
2. Magnetic resistance effect method. This method utilizes the changing characteristics of material resistances under magnetic fields. The sensors with this effect mainly include semiconductor reluctance elements and ferromagnetic thin film reluctance elements [55,56].
3. Hall Effect method. The electromotive force is generated by the electric current in the magnetic field. The change of the magnetic field intensity can be obtained by measuring the electromotive force. Figure 10 shows a photo of a magnetic pig with the Hall sensors. Compared with other measurement components, the Hall component manufacturing process is mature, and the stability and the temperature characteristics are better, so it is the first choice for leakage magnetic field measurement. In order to improve the measurement coverage and control resolution and prevent undetected flaws, more pieces of the Hall element will for man array to compose a leakage magnetic probe according to a certain pattern. Multi-channel data acquisition is available in order to improve the clarity of pipeline defect detection. Finally in order to prevent the occurrence of angle deflection of a Hall element on the circuit board due to collision, vibration and other incidents, the package is encapsulated, which can not only ensure the detection sensitivity and accuracy of the leakage magnetic probe, but also guarantee the strength and reliability of the wire and the circuit board connection [57,58,59].

**Figure 10:**
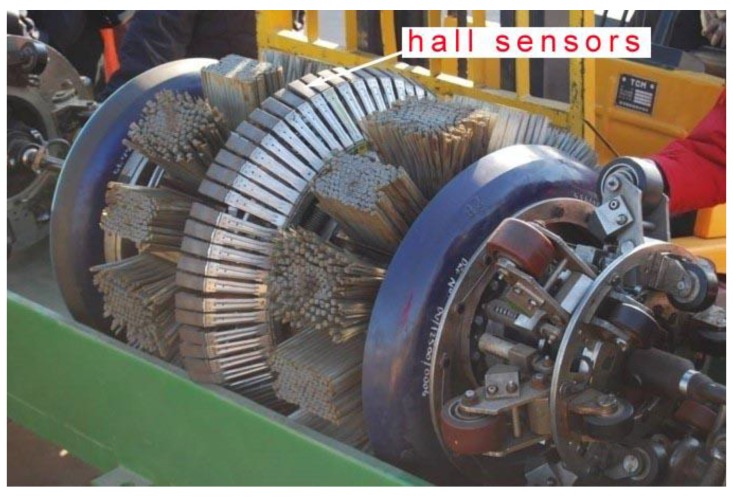
Photo of the magnetic pig with the Hall sensors.
4. Magnetic resonance imaging. By absorbing or radiating a certain frequency of electromagnetic wave in the magnetic field, some microscopic particles would induce resonance. The intensity of the magnetic field is obtained by measuring the degree of resonance. Due to different types of particle resonance, devices can be of various types, such as nuclear magnetic resonance magnetometers, electron spin resonance magnetometers, *etc*. The former is a measuring instrument with a constant magnetic field of the highest precision, so it can be used as a reference for magnetic transmission devices [60].
5. Magneto optical method. This approach utilizes the magneto-optical and magneto-stricture effects. Optical fiber sensors based on this method have unique advantages, which include being usable in harsh environments.

The magnetic sensor is a device that transforms the magnetic signals into electrical signals. There are many kinds of magnetic sensors, including induction coils, Hall components, magnetic flux gates, magnetic sensitive diodes and transistors, magnetic resistances [61], *etc.* The measurement ranges of different magnetic sensors can be seen in Table 1.

**Table 1:** Measurement ranges of magnetic sensors.

Magnetic Sensor	Measurable Intensity/T
Induction coils	10^−13^~10
Hall components	10^−5^~10
Magnetic flux gate	10^−12^~10^−13^
Magnetic sensitive diode	10^−6^~10^−1^
Magnetic sensitive transistor	10^−6^~10^−1^
Magnetic resistance	10^−11^~10^−3^

1. Induction coils. When the coils move on the surface of the pipe, the leakage magnetic field caused by a defect can cause a change of the magnetic flux through the coil. The induced electromotive force generated by the magnetic leakage field can be expressed by the following formula:
  (1) $$V=N\frac{d\emptyset }{dt}=N\frac{d\left(B\cdot S\right)}{dt}$$
  where N refers to the number of the coils; ∅ refers to the magnetic flux leakage flux in the coils; B refers to the magnetic induction intensity; S refers to the cross-sectional area of the coils. The relative variation of the magnetic field is measured by the induction coil, which is sensitive to high frequency signals. The sensitivity depends on the number of coils and the relative movement speed, and it is easily influenced by the speed of the coil movement.
2. Hall components. When the current movement direction is perpendicular to the direction of the magnetic induction intensity [62], Hall components on both sides produce a Hall electromotive force. It can be expressed by the following formula:
  (2) $$V_{H}=K_{H}\times I\times B\times \text{cos}\alpha$$
  where V_H_ refers to the Hall electromotive force; K_H_ refers to the Hall coefficient; I refers to electric current; B refers to the magnetic induction intensity; cosα refers to the normal angle between magnetic induction intensity and Hall components. The term V_H_ has nothing to do with motor speed, so it is not affected by the non-uniformity of the pipeline inspection.
3. Magnetic flux gate. The typical flux gate generally has three windings: drive winding, output windings and control windings. It is usually used to measure weak magnetic fields; the output depends on the magnetic properties of the magnetic core, and the resolving power varies with magnetic core and coil size. In recent years, some scholars have used amorphous alloys as magnetic cores, and sensitivity was greatly increased [63].
4. Magnetic sensitive diode and transistor. A magnetic sensitive diode is a new type of magnetoelectricity conversion component. Its sensitivity is high, and it is suitable for detecting small magnetic field changes. Its working voltage and sensitivity decreases with the increase of temperature so that compensation is needed. The magnetic sensitive transistor is a new type of semiconductor transistor which is sensitive to magnetic fields. It can be divided into NPN type and PNP type. Both of them have high sensitivity, but because of the nonlinearity of the temperature coefficient and the output, few have been applied in fact [64].
5. Magnetic resistance. The sensitivity of magnetic resistance is about 20 times that of the Hall component. Typically is 0.1 V/T, and its working temperature range is −40 to 150 °C. Its spatial resolution is related to the sensing area of the element.

The non-magnetic state sensors mainly include [65,66,67]:

1. A detector consisting of two odometers, where the output signal of the actual operation always uses two odometers with the fastest running being a mileage wheel as the system trigger signal, which can avoid failures caused by a mileage wheel.
2. Pressure sensors are used to measure absolute hydraulic or pneumatic pressure. Since the pressure inside the pipe also affects the leakage magnetic field, it is necessary to understand the pressure conditions within the pipeline.
3. A differential pressure sensor detects the pressure difference of the transmission medium before and after the skin bowl. It provides an auxiliary parameter for the operating speed of the detector.
4. A temperature sensor which uses the thermocouple principle is used to detect the internal temperature of the pipe.

### 3.2. Detection Signal Processing Method

**Figure 11:**
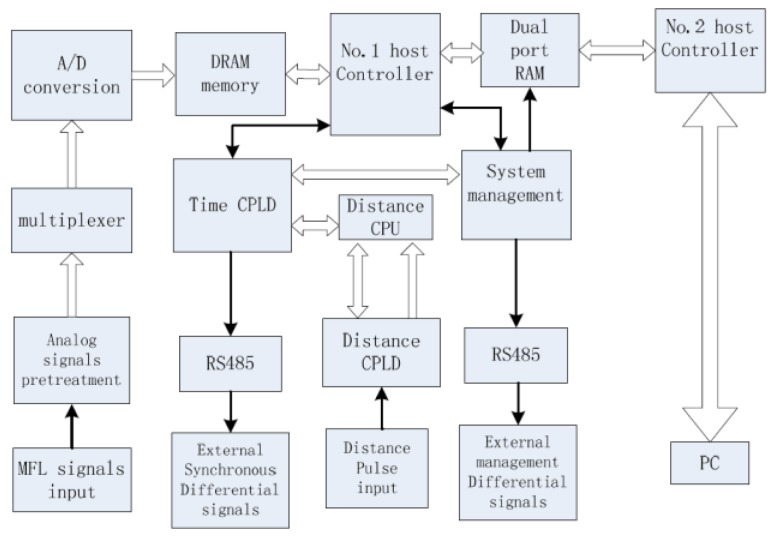
Diagram of the high-speed data collection system structure.

The processing of the detection signal includes data acquisition, storage and compression and noise reduction. Figure 11 shows a kind of high-speed data collection system structure. The data collected are magnetic data and non-magnetic data. Magnetic data is the leakage magnetic data of the pipeline defect detected by the magnetic sensor, which accounts for most of the data obtained by the detector. Depending on the size of the pipeline diameter, the number of sensors required will also change accordingly [68,69].The non-magnetic data include the working state of the detector, the speed of the detector, the position in space, the tube pressure, the pipeline temperature, *etc.* The non-magnetic data required by the speed relative to the magnetic data is lower. Data storage uses SATA standards, LABVIEW programming is used to write the related configuration page [70,71,72].

Since the system itself can only provide limited storage capacity and storage speed, it is necessary to compress huge amounts of data. In general, most of the detection data is a small signal in the whole detection process, and only in the vicinity of the defect will a large signal appear. The core idea of segmentation threshold compression algorithms is that in the place where the original amplitude of the signal is relatively large little or no compression can be used, and in the smaller amplitude areas large compression ratios are chosen. Noise reduction is needed to improve the signal quality and increase the accuracy of data analysis [73,74].

### 3.3. Ground Marking Method

**Figure 12:**
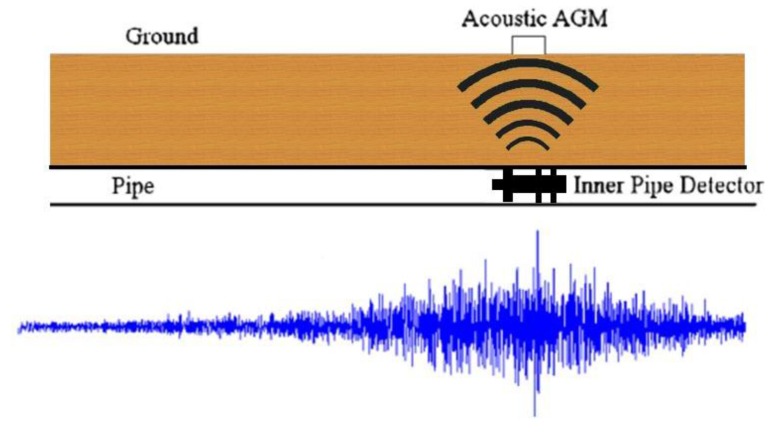
Signal acquired by an acoustic AGM when an inner pipe detector is approaching.

As one of the three major components of Magnetic flux leakage, an above ground marking system can be used to locate and assess the integrity of long distance pipelines. When a Magnetic flux leakage detector is moving in the pipeline, it detects the cracks and records the mileage value [75,76,77,78]. The odometer value is particularly important, because it records the location of defects and provides reference data for future pipeline excavations. However, the odometer will have accumulated errors, which are caused by the mechanical structure or detector rotation and sliding and other reasons. According to the statistics, when the pipeline equipment moves 1km, the cumulative error can reach 1m, and the error will be accumulated over the whole detection process. As is known to us, long pipelines can reach lengths of tens of thousands of kilometers, so if there is no compensation method, the positioning error may reach several tens of meters. Since a positioning error of up to a few meters will lead to huge mining work, the errors must be corrected. High-precision above ground marking systems can be used to resolve this problem. In general, the hardware part of above ground marking system is composed of Master Clock system and Above Ground Marker (AGM). The PIG detects the wall defects of the pipe and records the defect position through the mileage wheel, providing support for the excavation and maintenance of the pipeline. Pollutants in the wall can cause wheel slips and then generate an accumulated error, so it must be calibrated by other means so as to realize the precise positioning of the defects. The main positioning technologies are as follows [79,80,81]:

- Odometer positioning technology is one of the earliest positioning techniques. However, due to the presence of oil defects and other causes in the pipeline, the odometer will slip resulting in cumulative errors, therefore, this technology alone can’t meet the accuracy demands of positioning.
- Static magnetic field positioning technology uses permanent magnets and Hall elements for location. When the PIG runs through the positioning device, magnetic sensors will receive the changing magnetic field. By judging the received signal peak volatility, the location of the detector can be determined.
- Ultra-low frequency electromagnetic field positioning technology is the most widely used technology. Figure 12 gives a common example. Because of the extremely low frequency of the electromagnetic field, the electromagnetic field has good penetration through metal, soil and air, so it is widely used in the detection of defects in oil/gas pipelines.

### 3.4. Quantitative Analysis of the MFL

The analysis and identification of the leakage magnetic signal after any compensation is the key of the quantitative analysis of the magnetic flux leakage detection [82,83,84,85]. The defect appearances are derived from the magnetic flux leakage signals, including the distinction between different kinds of defects and to obtain the characteristic information of qualitative analysis and the defect shape parameters to make quantitative analysis, can be attributed to a typical inverse electromagnetic field problem. The inverse electromagnetic field problem can be divided into two categories: the optimal design problem and the parameter identification problem [86,87,88,89,90]. The former is also known as a comprehensive problem. The difference between the two is that the optimization design problem is generally not the uniqueness of the solution, but the existence of the solution is required. The problem of parameter identification is the only solution to the objective reality.

In view of the characteristics of the inverse problem of magnetic flux leakage detection, the method can be divided into direct methods and indirect methods. Because of the great difficulty of solving the direct method, people have put forward various indirect methods to solve the defect parameters based on the detection signal approximation. These methods can be divided into three categories: mapping methods, iterative methods and signal classification methods. 

The mapping method is also called the pattern matching method, according to the present commonly used algorithm; the method can be divided into statistical-based and neural network. The two mapping methods mentioned above have a common defect that places overreliance on the consistency and accuracy of the statistical sample and the training samples, and a lack of expansion, the defects of complex structure and quantization accuracy to detect the actual shape is low; another important question is that the existing mapping methods are built on the basis of analysis of two-dimensional problems [91,92,93]. Although the two dimensional method can simplify the study, it can help to understand the change of the defect signal. In fact, the leakage magnetic signals and defect dimensions are nonlinear, so there is a more complex variable function and in the actual detection, the defect of 3D shape parameters are unknown.

The iterative method is widely used in solving the inverse electromagnetic field problem. The essence of this method is to solve the positive problem and solve the inverse problem in the feedback way. It first needs to estimate the defect parameters, solving the corresponding forward problem, and this gives the distribution of the leakage magnetic field; then the measured values are compared, and if the error is greater than a predetermined threshold, the defect parameters are adjusted for the calculation. The process should be iteratively repeated until the error value is less than a predetermined threshold.

The signal analysis method, also called the pattern classification method, involves dividing the inverse problem into a limited number of defects, including clustering algorithms and neural network methods. At present, neural networks have been widely used in the classification of eddy current testing and ultrasonic testing signals.

In summary, the direct method cannot obtain a unique and stable solution because of the ill posed problem of the electromagnetic field inverse problem. In the indirect method, the mapping method is widely used, but it needs a large number of sample data. The existing methods mainly depend on the single leakage magnetic field characteristics and defect parameter mapping, without considering the actual existence of a much more complex variable function, so the error is large, and it cannot meet high precision requirements; the computational efficiency of the iterative method is too low; signal classification, although widely used, but since it only provides the realization of signal classification, it is unable to achieve the accuracy of quantitative parameters and meet the requirements of high precision detection [94,95].

Before the identification of the defect leakage signals, the magnetic flux signal is firstly processed, which includes the recognition of foreign body signals and interpolation of the waveforms. Then the feature signal is needed. To facilitate the analysis, signal waveform characteristics should be defined, such as: wash the wave trough value, signal threshold value interception length, waveform area and so on. After that, a premade standard defects library can be used. Keeping other defect dimensions (length, width or depth) unchanged and according to the change of signal, the closest relationship with characteristic signals of the flaw size could be filtered out and then statistical recognition can be carried out.

### 3.5. Statistical Identification of Defect Length, Width and Height

The relationship between the length of the defects and the span of the leakage field is approximately linear, and the defect length is also related to the defect depth, because the leakage magnetic field is related to the depth of the defect [96,97,98]. Therefore, any uniform threshold used to quantify the length will contain obvious errors. A dynamic threshold can be used to eliminate the impact of depth.

In the quantitative analysis of the defect, width and depth are associated with the multiples of signals respectively. There are nonlinear relationships between these characteristics, which constitute a typical multivariable statistical analysis; the solution of this problem can be collected by multiple nonlinear regression methods, principal component analysis, linear pattern classifiers, nonlinear discriminator functions and other statistical methods.

The width of a defect in quantitative analysis mainly depends on the circumferential span of the defect leakage magnetic field of the pipe, and quantization precision depends on the distribution of the probe and the distance between the circumferential probes.

The quantitative discrimination of the width is mainly dependent on the wave signal of the circumferential distribution of the leakage magnetic field, and the obvious characteristics of the signal includes [99,100]: the circumferential leakage magnetic field waveform signal peak value, circumferential waveform signal intercept length threshold, number of leakage magnetic fields to cover the probe channel and number to cover defects of the leakage magnetic field differential probe. A dynamic threshold is still needed for the quantification of the width.

The evaluation of defect depth has been a difficult problem in pipeline corrosion detection. The flaw depth is most obvious with the axial peak and vale of the magnetic leakage field, because it is the largest among all the defects, but the peak and valley values are affected by the length and width of the defect. Therefore, the evaluation of the depth of the defect should include the feature characteristics of width and length in the magnetic flux leakage field [101,102].

In summary, using statistical methods for evaluation of the defect shape parameters (length, width and height) is a complex process, where the evaluation of the length of the most easily accomplished; the evaluation of the width and depth is more complicated. The statistical method is verified by field practice, and the length quantitative results can reached up to 90% accuracy, the width quantitative results can reach 84% accuracy, and the depth quantitative results can reach 78% [103,104,105].

Although statistical recognition methods for characterizing defects has achieved good results in practice, statistical methods don’t have automatically adaptive processing ability, and this method requires large volumes of testing or simulation data, and the statistical process is relatively complex. In order to improve the accuracy of detection and improve the level of intelligence, many scholars at home and abroad have used neural network technologies to analyze the data. A neural network is a network composed of a large number of processing units, which reflects the basic features of human brain function, and is a kind of abstraction, simplification and simulation of the human brain. The information processing function of the network is realized by the interaction between the neurons. The storage of knowledge and information acts as the physical contact between the network components [105,106,107].

Nowadays the neural network method has been successfully applied to processing magnetic flux leakage signals and leakage magnetic field reconstruction; but in the pipeline leakage magnetic detection for quantitative analysis, the application of neural networks is still in the initial stage. The existing methods, on the one hand, are slower to calculate and the degree of recognition accuracy is low; on the other hand, it can only analyze the existing test results and cannot accurately identify arbitrary defects.

The multivariate interpolation algorithm of radial basis function neural networks originated from numerical analysis of a former non-feedback neural network. The network has the function of being the approximation of the functional approximation and the optimal functional approximation property [108]. Its other advantage is that it has a fast convergence rate, and is suitable for the approximation of the multivariable function. As long as the center location is properly chosen, only a few neurons can give a good approximation effect.

Compared with the ordinary neural network method, the proposed method can improve the adaptability of different defects through the closed-loop structure, and it also has the advantages of fast convergence speed, strong anti-noise properties and so on.

### 3.6. 3D Finite Element Neural Network Method

**Figure 13:**
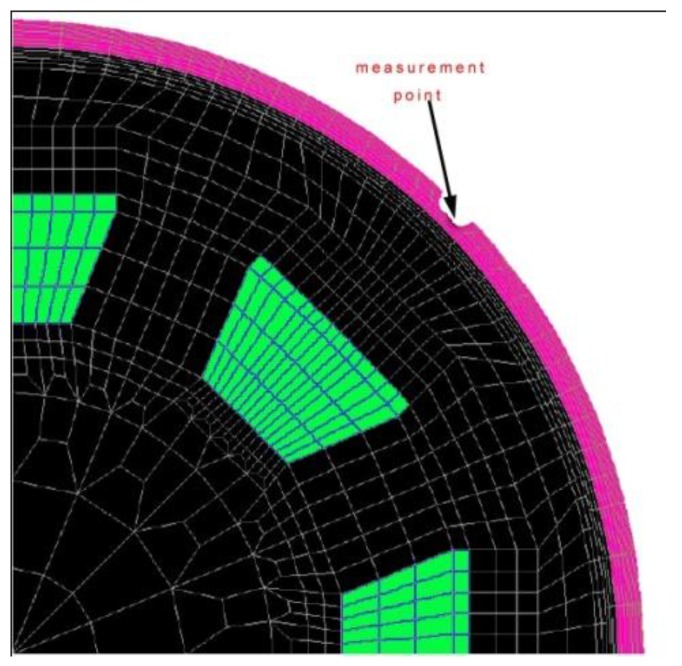
The measurement point used for a central defect, for a 0.625 inch thick, 30 inches OD pipe. The measurement position is 8 mm from the inside of the pipe wall.

The main advantage of the 3D finite element analysis with neural networks is that its essence is a parallel implementation of the finite element calculation model, so as to be convenient for hardware and software based on the realization of parallel computing, which can greatly accelerate the speed of finite element calculations. Figure 13 gives a three-dimensional diagram based on the finite element method.

Another important advantage is that because most of its weights are calculated and stored in advance, there is no need for any training. Because the weight depends on the problem of the differential equation and boundary conditions, as long as the two points do not change, the weight will not need to change. This means that in the use of the FENN as a quantitative method of corrosion defects, there is no dependence on training samples, so it can improve ability to detect irregularly shaped defects. One of the main disadvantages is that it needs lots of neurons, which makes it more demanding of system memory capacity.

## 4. Expert System

Expert systems are one of the important aspects of artificial intelligence work. Their core is the representation of human knowledge and experience. In engineering applications, they give analysis results according to the knowledge and experience of experts in the field, hence it is called the expert system. Before the leak magnetic detection intelligent quantization scheme is put forward, the analysis and evaluation of the magnetic flux leakage data is given by the experts or senior technical personnel in the field [109,110]. Until now, although a lot of scholars have done a lot of research on magnetic flux leakage detection, the evaluation of the risk of defects still very much relies on experience. In order to facilitate the engineering application, these experiences are summarized as different evaluation criteria by the engineering technical units of various countries.

Although there is no uniform, accurate, generally accepted definition, according to the views of scholars in related fields, there are some common parts in an expert system. If the function of the system is considered, it should have the following functions:

Knowledge base which is the knowledge required to store the solution to the problem can be understood as a rule to be followed. Different types of knowledge representation methods and different representation methods are a research hot spot is this area [111].

Comprehensive database which used to store the various information involved in the initial data and reasoning process, such as intermediate results, objectives, assumptions, *etc.*

Inference engine which can solve the target problem according to the current data input, the use of existing knowledge and a certain reasoning strategy. It can control and coordinate the whole system. The main reasoning strategy is divided into three kinds: forward reasoning, backward reasoning and hybrid reasoning.

The interpreter is capable of providing the necessary explanation of the process, conclusions, or the behavior of the system itself [112,113].

Knowledge acquisition modules, providing channels for knowledge acquisition, allow the knowledge base to be modified and expanded, making it possible for the system to grow which can improve the problem solving ability and the accuracy of the system.

Human-computer interaction interface can provide a user interface, which is convenient for users to use and understand, and it can facilitate analysis and understanding of the various users’ requests.

## 5. Future Developments

In short, the current status of research is that some gratifying results in theoretical and experimental studies have been achieved, but there is still no complete theoretical system; more qualitative analysis exists but the engineering using quantitative research work is less frequent; the defect contour description is still in the stage of laboratory research, and actual pipeline defect detection accuracy still needs to be further improved.

Pipeline detectors are evolving towards higher resolution, higher precision and high positioning accuracy. GPS, pipeline direction system and automatic speed control systems and other auxiliary devices will greatly improve their technical performance. At the same time, due to the development of computer and image processing technology, the accuracy of the detection results and the description of the defects will be greatly improved. Pipeline inspection technology will also be combined with other technologies such as ultrasonic testing. The intelligent interpretative combination of the two ILI information sources significantly exceeds the straightforward statistical combination benefits, thus reducing follow-up costs for the pipeline operator; three axis high definition magnetic flux leakage detectors which could record three independent directions of the magnetic leakage signals and more clearly geometrical characteristics of the defect would be described could be put into practical use; dual field magnetic flux leakage technology will be more mature, which will allow for more accurate detection identification and characterization of features that are accompanied by residual stresses such as dents; pipeline operators will also utilize robotic inspection tools with NDE systems, capable of negotiating the obstacles in the pipeline under low pressure and flow condition without human effort; UAV technology will also be applied to achieve better positioning of the detector; expert systems will be more intelligent, with a strong self-learning and evolution ability, and will be able to provide more accurate solutions to common problems.

## 6. Conclusions

This paper introduces the principle, measuring methods and quantitative analysis of the magnetic flux leakage (MFL) method:

1. MFL is the most popular method of pipeline nondestructive testing technique which uses a magnetic sensitive element to detect the defects on both internal and external surfaces. Application of numerical calculation methods has made great progress in the theoretical research, and the relationship between the shape of the defect and the leakage magnetic field is well established. But there are many limitations when put into the practice.
2. Because of their mature manufacturing process, favorable stability and temperature characteristics, Hall sensors are the first choice for measurement of leakage magnetic fields.
3. The processing of the detection signal includes data acquisition, storage and compression and noise reduction.
4. Ground marking systems are used to determine the position of the detector and facilitate pipeline excavation.
5. Statistical identification methods are used to establish the relationship between the defect shape parameter and magnetic flux leakage signals. A 3D finite element neural network is convenient for hardware and software based on the realization of parallel computing.
6. Expert systems are an important aspect of artificial intelligence work. They gives the analysis result according to the knowledge and experience of the experts in the field, but nowadays the evaluation relies on too much this experience.
